# Systematic multi-parameter optimization of an injectable thermosensitive CS/β-GP/HEC hydrogel for 3D cell culture applications

**DOI:** 10.1371/journal.pone.0354590

**Published:** 2026-07-29

**Authors:** Wenjing Zhao, Ying Liu, Jiali Yao, Zhenlu Zhou, Linlu Yan, Hao Liang, Qiping Hu

**Affiliations:** 1 School of Basic Medical Sciences, Guangxi Medical University, Nanning, Guangxi, China; 2 The First Affiliated Hospital of Guangxi Medical University, Nanning, Guangxi, China; University of the Witwatersrand, SOUTH AFRICA

## Abstract

Injectable thermosensitive hydrogels have been widely investigated as minimally invasive scaffolds for three-dimensional (3D) cell culture. In this study, we systematically screened a chitosan (CS)/β-glycerophosphate (β-GP)/hydroxyethyl cellulose (HEC) ternary hydrogel system using a 2 × 2 × 2 factorial design. By evaluating gelation kinetics, injectability, and structural stability through a weighted scoring model, two superior formulations (G1 and G3) were selected for their ability to balance rapid gelation at 37 °C with reliable room-temperature injectability. Subsequent biological evaluations demonstrated that, compared to conventional 2D cultures, the 3D hydrogels supported robust cellular viability and spatial proliferation through Day 10. The stable biomimetic matrix provided essential physical anchorage, enabling Huh7 cells to self-assemble into dense tumor spheroids and BMSCs to establish spatial networks. This study presents a structured formulation-screening strategy and demonstrates the effectiveness of the CS/β-GP/HEC hydrogel as a reliable platform for *in vitro* 3D cell culture applications.

## Introduction

In recent years, injectable thermosensitive hydrogels have been extensively investigated for applications in tissue engineering and regenerative medicine due to their unique ability to undergo sol–gel transitions upon injection, providing minimally invasive, biomimetic scaffolds for cells and drugs [1]. Recent advances have highlighted the importance of adaptive mechanical properties in these scaffolds for guiding tissue remodeling, ensuring that the matrix can respond to cellular cues during the healing process [2]. Their appeal lies in their ability to be injected at room temperature, undergo rapid gelation *in situ*, and conformally fill irregular defects, while maintaining thermal stability and predictable degradation behavior—critical factors for the clinical performance of polysaccharide-based systems [3]. Among the various systems, the chitosan (CS)/β-glycerophosphate (β-GP) system has garnered attention for its ability to undergo physical crosslinking under physiological conditions without chemical crosslinkers [4,5]. However, classical CS/β-GP systems often exhibit insufficient mechanical strength, motivating the development of multicomponent formulations [6,7].

To address these limitations, hydroxyethyl cellulose (HEC) has been incorporated into CS/β-GP systems to form ternary hydrogels, leveraging multicomponent synergy to enhance network stability. The thermoresponsive transition is governed by hydrogen bonding and hydrophobic interactions [8]. The addition of HEC further modulates rheological behavior and pore microstructure, creating microenvironment-responsive platforms that can adapt to dynamic biological environments [9]. Previous studies suggest that gelation strength and kinetics can be tuned for specific applications, such as pancreatic tissue engineering [10]. Moreover, the CS/β-GP/HEC system is generally biocompatible and can mimic extracellular matrix (ECM)-like microenvironments to support cell adhesion and three-dimensional (3D) growth [11–13].

Notably, while many studies focus on optimizing individual components, a critical gap remains in understanding how the synergistic interactions between CS viscosity, β-GP concentration, and HEC viscosity dictate the high-order spatial organization of encapsulated cells. For 3D cell culture, a scaffold must provide not only operational feasibility but also a specific mechanical niche and architectural cues that support cellular remodeling. Therefore, this study employed a 2 × 2 × 2 factorial design to systematically compare eight CS/β-GP/HEC formulations. Beyond screening for gelation and injectability, we utilized dynamic rheology and advanced cytoskeletal imaging to evaluate how optimized candidates (G1 and G3) influence the 3D proliferation and morphological adaptation of Huh7 cells and bone marrow-derived mesenchymal stem cells (BMSCs). By bridging the gap between polymer rheology and 3D cellular spatial organization, this work provides a methodological framework for tailoring biomimetic scaffolds for diverse tissue engineering needs.

## Materials and methods

### Reagents and instruments

Chitosan (CS; low viscosity: 100–200 mPa·s, Cat. No. C105799; high viscosity: 200–400 mPa·s, Cat. No. C105802), β-glycerophosphate disodium salt (β-GP, Cat. No. D106347), hydroxyethyl cellulose (HEC; low viscosity: 2600–3300 mPa·s, Cat. No. H104793; high viscosity: 3400–5000 mPa·s, Cat. No. H810925), and glacial acetic acid (analytical reagent grade, 99.5%) were all purchased from Shanghai Aladdin Biochemical Technology Co., Ltd. (Shanghai, China). Optimal Cutting Temperature (O.C.T.) compound was obtained from Sakura Finetek Japan Co., Ltd. (Japan). A Live/Dead Cell Staining Kit was sourced from APExBIO (USA). Huh7 cells were acquired from the Cell Bank of the Chinese Academy of Sciences (Shanghai).

### Preparation and grouping of CS/β-GP/HEC thermosensitive hydrogels

(1) CS solution (2% w/v): CS powder (0.5 g) was autoclaved and dissolved in 25 mL of a 1% (v/v) acetic acid solution.
(2) β-GP solutions (5.6% and 36% w/v):β-GP powder (0.56 g or 3.6 g) was sterilized under UV light for 60 minutes and dissolved in 10 mL of low-glucose Dulbecco’s Modified Eagle Medium (L-DMEM).
(3) HEC solution (2.5% w/v): HEC powder (0.25 g) was sterilized under UV light for 60 minutes and dissolved in 10 mL of L-DMEM.
(4) The ternary thermosensitive hydrogel system was prepared by mixing CS, β-GP, and HEC solutions at a fixed volume ratio of 8:3:2.5, respectively [6,14,15]. Eight experimental groups were designed by varying CS viscosity, β-GP concentration, and HEC viscosity, as detailed in Table 1.

**Table 1:** Formulation design of the eight CS/β-GP/HEC systems (CS:β-GP:HEC = 8:3:2.5, v/v/v).

Group	CS (mPa·s)	β-GP (% w/v)	HEC viscosity (mPa·s)	Factor code
1	200–400 mPa·s	36	2600**–**3300 mPa·s	CS-H/ βGP-H/ HEC-L
2	200**–**400 mPa·s	36	3400**–**5000 mPa·s	CS-H/ βGP-H/ HEC-H
3	200**–**400 mPa·s	5.6	2600**–**3300 mPa·s	CS-H/ βGP-L/ HEC-L
4	200**–**400 mPa·s	5.6	3400**–**5000 mPa·s	CS-H/ βGP-L/ HEC-H
5	100**–**200 mPa·s	36	2600**–**3300 mPa·s	CS-L/ βGP-H/ HEC-L
6	100**–**200 mPa·s	36	3400**–**5000 mPa·s	CS-L/ βGP-H/ HEC-H
7	100**–**200 mPa·s	5.6	2600**–**3300 mPa·s	CS-L/ βGP-L/ HEC-L
8	100**–**200 mPa·s	5.6	3400**–**5000 mPa·s	CS-L/ βGP-L/ HEC-H

Formulations were prepared at a fixed volume ratio of CS:β-GP:HEC = 8:3:2.5 (v/v/v). The β-GP percentages refer to stock solution concentrations prior to mixing.

Eight experimental groups were prepared according to a 2 × 2 × 2 factorial design by varying CS viscosity, β-GP concentration, and HEC viscosity (detailed in Table 1). These formulations were designated as G1–G8, which are used consistently throughout the manuscript and figures. Specifically, G1 and G3 correspond to the high-β-GP (36%) and low-β-GP (5.6%) formulations, respectively.

### Adjustment of pH and thermogelation conditions

The initial pH values of the individual solutions were measured as follows: CS solution, pH 6.5; β-GP solutions (5.6% and 36%), pH 8.0; and HEC solutions, pH 8.0. Notably, upon mixing these components in an ice bath, the resulting precursor hydrogel solution (sol state) achieved a physiological pH range of 7.2–7.4 prior to gelation. This neutralization is primarily driven by the alkaline β-GP, which ensures cytocompatibility for subsequent cell encapsulation. After mixing and confirmation of physiological pH, the samples were incubated at 37 °C [16]. A fixed incubation period of 25 minutes was employed to allow stable gel formation; the methodology for determining the precise gelation time is detailed in Section 2.4.

### Gelation behavior, gelation time, and injectability

(1) Gelation assessment: Flowability at 25 °C and gel formation at 37 °C were monitored visually. Gelation was defined as the point at which the sample exhibited no flow upon inversion of its container [16,17].
(2) Gelation time (*tgel*): The mixtures were incubated at 37 °C, and the time required to reach the “no flow upon inversion” state was recorded as the gelation time. Measurements were performed in triplicate for each sample group (n = 3), and results are presented as mean ± standard deviation (SD).
(3) Injectability: Injectability was assessed using a 1 mL syringe fitted with a 26G needle (inner diameter: 0.45 mm) at an injection rate of 0.1–0.5 mL/min. The evaluation criteria included smooth extrusion of the formulation and the absence of needle clogging or partial clogging [16]. Formulations were classified as injectable, partially clogged, or clogged based on whether continuous extrusion could be maintained without a marked increase in manual injection force.

### Rheological characterization

The dynamic rheological properties of the selected hydrogel formulations (G1 and G3) were evaluated using a rotational rheometer (Haake Mars 40, Thermo Fisher Scientific, Germany) equipped with a parallel-plate geometry (20 mm diameter). A solvent trap was employed to prevent evaporation during measurements. The experiments were conducted as follows:

(1) Temperature Sweep Test: To determine the sol-gel transition temperature (*T*_*gel*_), the hydrogel precursor (pre-cooled to 4 °C) was loaded onto the Peltier plate. The temperature was ramped from 20 °C to 50 °C at a constant heating rate of 1 °C/min. The storage modulus (*G*′) and loss modulus (*G*′′) were recorded at a constant frequency of 1 Hz and a strain of 1%. The strain amplitude was predetermined via strain sweep tests to ensure measurements were within the linear viscoelastic region (LVR). *T*_*gel*_ was defined as the crossover point of *G*′ and *G*′′.
(2) Time Sweep Test: To quantify the gelation kinetics at physiological temperature, samples were rapidly heated to 37 °C and held isothermally. The evolution of *G*′ and *G*′′ over time was monitored under constant frequency (1 Hz) and strain (1%).
(3) Shear Rate Sweep Test: To validate injectability, the steady-state viscosity of the precursors was measured at 25 °C over a shear rate range of 1–1000 s⁻^1^, demonstrating the shear-thinning behavior of the formulations prior to gelation.

### In vitro mass loss assay

Hydrogels (1.68 mL per well) were prepared in six-well plates. Each well was then supplemented with 2 mL of L-DMEM and incubated at 37 °C. The culture medium was replaced daily. Prior to each medium change, the gels were carefully removed, gently blotted to remove excess surface liquid, weighed, and photographed. Each experimental group consisted of three replicate wells (n = 3).

The percentage mass loss was calculated using the following formula: Mass loss (%) = [(Initial mass − Mass at time t)/ Initial mass] × 100. Data are presented as mean ± standard deviation (SD; n = 3). Statistical comparisons among groups are presented in Fig 3.

### Morphological characterization

(1) Light microscopy: The micro-morphology of the hydrogels was observed using an inverted phase-contrast microscope (Leica DMi1, Leica Microsystems, Germany). Observations were completed within 15 minutes after removing samples from the CO_2_ incubator to preserve the structural integrity and cellular viability of the constructs.
(2) H&E staining: For histological analysis, the cell-scaffold constructs were fixed in 4% paraformaldehyde for 24 h, embedded in O.C.T. compound, and then prepared into frozen sections. The sections underwent graded ethanol dehydration (80%–100%), followed by staining with hematoxylin (5 min), differentiation in 0.5% hydrochloric acid-alcohol (10 s), and counterstaining with eosin (10 s) to visualize the porous architecture.
(3) Scanning electron microscopy (SEM): Hydrogel samples were rapidly frozen in liquid nitrogen (−196 °C) for 15 min and lyophilized for at least 10 h. The freeze-dried samples were sectioned, and the cross-sections were mounted upwards on conductive adhesive. After sputter-coating with gold for 30 s, the network topology and pore morphology were examined using a scanning electron microscope (VEGA3 LMU, TESCAN, Czech Republic) at an accelerating voltage of 30.0 kV and a working distance of 15.17 mm. The images presented are representative of the observed morphology.

### Cell culture and 3D encapsulation

Huh7 cells were purchased from the Cell Bank of the Chinese Academy of Sciences (Shanghai, China) and maintained through routine subculture. BMSCs were isolated from the bone marrow of 7-day-old newborn Sprague-Dawley (SD) rats according to established protocols [18]. All animal procedures were approved by the Institutional Animal Care and Use Committee of Guangxi Medical University (approval period: January 1, 2023, to December 31, 2026). Prior to 3D encapsulation, BMSCs at passage 3 (P3) were authenticated via phenotypic surface markers and multi-lineage differentiation capacity to confirm their stemness. Log-phase cells were harvested, counted, and resuspended in pre-cooled CS solution to prepare a cell suspension. To achieve a final cell density of 1 × 10^5^ cells/mL within the hydrogel, the cell suspension was adjusted to a concentration of approximately 6.6 × 10^5^ cells/mL. Specifically, adding 2 mL of this cell suspension to a total mixture volume of 13.17 mL yields the desired final density.

All subsequent steps were performed under cold conditions to prevent premature gelation. The cell-loaded precursor mixture was prepared by sequentially combining the following cold components: 6.67 mL of CS solution, 2.50 mL of β-GP solution, 2.00 mL of HEC solution, and finally 2.00 mL of the prepared cell suspension, resulting in a total volume of 13.17 mL. The mixture was gently homogenized to avoid bubble formation. For the characterization of blank (acellular) hydrogels, a base volume ratio of CS:β-GP:HEC = 8:3:2.5 was used. Since the cells were resuspended in 2% (w/v) CS, the 2 mL cell suspension was considered part of the CS phase. To maintain a consistent formulation rationale, the cell-loaded recipe can be described as: CS 6.67 mL + β-GP 2.50 mL + HEC 2.00 mL + cell suspension (in 2% CS) 2.00 mL. The formulation proportions remained consistent with the predefined base ratio. The final concentrations within the cell-laden gels were: CS 1.32% (w/v) and HEC 0.38% (w/v). The β-GP final concentration was 1.06% (from a 5.6% stock) or 6.83% (from a 36% stock). Preliminary tests confirmed that the inclusion of the cell suspension and the slight adjustment in volume ratios did not significantly alter the macroscopic gelation time compared to the cell-free formulations.

The precursor mixture was dispensed into six-well plates (2 mL per well) and allowed to gel at 37 °C under 5% CO_2_ for 15–20 minutes. Subsequently, low-glucose Dulbecco’s Modified Eagle Medium (L-DMEM) was carefully overlaid. The culture medium was replaced every 48–72 hours, and cell morphology was observed daily. Huh7 cell encapsulation followed an identical procedure with the same final loading density of 1 × 10^5^ cells/mL (~2 × 10^5^ cells per 2 mL gel).

### Fluorescence cell staining (live/dead and cytoskeleton)

A working solution containing 2 µM Calcein-AM and 4.5 µM propidium iodide (PI) was prepared. Samples were washed three times with PBS, incubated with the working solution at room temperature in the dark for 60 minutes, rinsed again with PBS, and subsequently imaged using fluorescence microscopy. Staining was performed on days 7 and 10. For each group and time point, independent samples were used (n = 3). At least three random fields were acquired per sample for qualitative and, where applicable, quantitative analysis.

To evaluate cellular morphology and cytoskeletal organization, cell-laden hydrogels and 2D controls were harvested on day 7. Samples were fixed with 4% paraformaldehyde for 30 minutes, washed three times with PBS, and permeabilized with 0.1% Triton X-100 for 15 minutes at room temperature. Subsequently, the F-actin cytoskeleton was stained with TRITC-labeled phalloidin for 40 minutes in the dark, and nuclei were counterstained with DAPI for 10 minutes. Stained cells were visualized using an inverted fluorescence microscope (CKX41, Olympus, Japan). For each formulation, representative images were captured from multiple fields of view to evaluate the spatial distribution and morphological adaptations of the encapsulated cells..

### Cell proliferation assay

The proliferative activity of stably passaged BMSCs and Huh7 cells encapsulated within 3D hydrogels was quantitatively evaluated using a Cell Counting Kit-8 (CCK-8; Aladdin, China) assay, with standard 2D culture serving as a control.

Briefly, the hydrogel precursor solutions were sterilized under UV irradiation for 30 minutes. Cells were harvested, resuspended, and gently mixed with the pre-cooled (4 °C) precursors to achieve a final density of 1 × 10^5^ cells/mL. Subsequently, 100 μL of the cell-hydrogel mixture was injected into each well of a 96-well plate and incubated at 37 °C for 25 minutes to complete the phase transition. Following stable gelation, 100 μL of complete culture medium (L-DMEM supplemented with 10% FBS) was added. For the 2D control group, an equal number of cells (1 × 10^4^ cells/well) were seeded directly onto standard 96-well plates. The culture medium was refreshed every two days throughout the 10-day incubation period.

Cell proliferation was assessed at predefined time points (Days 1, 3, 5, 7, and 10). At each time point, the old medium was replaced with 110 μL of fresh medium containing 10% (v/v) CCK-8 working solution. After incubation in the dark at 37 °C for 1 hour, 100 μL of the supernatant from each well was transferred to a new 96-well plate to avoid interference from the hydrogel’s optical density. The absorbance was measured at 450 nm using a microplate reader (Multiskan K5800C, Thermo Fisher Scientific, USA). All experiments were performed in triplicate, and cell-free hydrogels treated with the same CCK-8 procedure were used as blank controls to eliminate background absorbance from the scaffold material.

### Weighted scoring evaluation for formulation selection

To quantitatively evaluate the overall suitability of the candidate formulations, a linear weighted scoring model was established. The total comprehensive score (*S*_*total*_) for each hydrogel candidate was calculated using the following formula [19]:


$$\begin{array}{c} S_{total}=\left(\text{0}.\text{6}\text{2}\times S_{gel}\right)+\left(\text{0}.\text{1}\text{8}\times S_{inj}\right)+\left(\text{0}.\text{2}\text{0}\times S_{stab}\right) \end{array}$$

where *S*_*gel*_ represents the gelation time score, *S*_*inj*_ represents the injectability score, and *S*_*stab*_ represents the structural stability score (derived from the mass loss rate). The weighting coefficients were defined a priori based on predefined operational priorities to ensure objective integration of handling and structural parameters. Specifically, gelation speed was assigned the highest priority (approximately 60%) to ensure rapid in situ fixation and prevent material diffusion, while injectability and stability were considered secondary yet essential supporting factors (approximately 20% each). The detailed evaluation parameters, individual calculated scores, and the final screening results for all candidate formulations are summarized in Table 2.

**Table 2:** Multi-objective scoring and screening results for candidate formulations.

Group	Gelation time, *t*_*gel*_ (min)	Gelation score, *S*_*gel*_	Injectability	Injectability score, *S*_*inj*_	Mass loss at 72 h (%)	Stability score, *S*_*stab*_	Total score, *S*_*total*_	Rank
1	10	1	Injectable	1	77	0.203	0.841	1
2	5	0.625	Clogged	0	75	0.000	0.388	4
3	17	1	Injectable	1	79	0.021	0.804	2
4	13	1	Partially clogged	0.5	83	0.437	0.737	3
5	＞30	0	Injectable	0	87	1.000	0.200	5
6	＞30	0	Injectable	0	78	0.334	0.067	8
7	＞30	0	Injectable	0	89	0.972	0.194	6
8	＞30	0	Injectable	0	85	0.521	0.104	7

*S*_*gel*_, *S*_*inj*_, and *S*_*stab*_ were calculated according to the scoring criteria described in Section 2.9. Formulations were ranked based on the total score (*S*_*total*_), with higher scores indicating better overall performance. Values exceeding the measurable range are indicated as “>30”.

### Statistical analysis

All quantitative data are presented as the mean ± standard deviation (SD). Statistical analyses and data visualization were performed using GraphPad Prism version 10 (GraphPad Software, Boston, MA, USA). All quantitative experiments were conducted with at least three independent replicates (n ≥ 3). Specific sample sizes for individual assays are detailed in the respective figure legends. One-way ANOVA with Tukey’s post hoc test was used for data with equal variances; Welch’s ANOVA with Dunnett’s T3 post hoc test was applied when variances were unequal. For two-group comparisons, two-sided independent-samples t-tests (or Welch’s t-test when appropriate) were employed. A p-value of less than 0.05 was considered statistically significant (p < 0.05). Exact p-values are reported where appropriate.

## Results

### Thermogelation behavior, gelation time, and injectability

**Fig 1:**
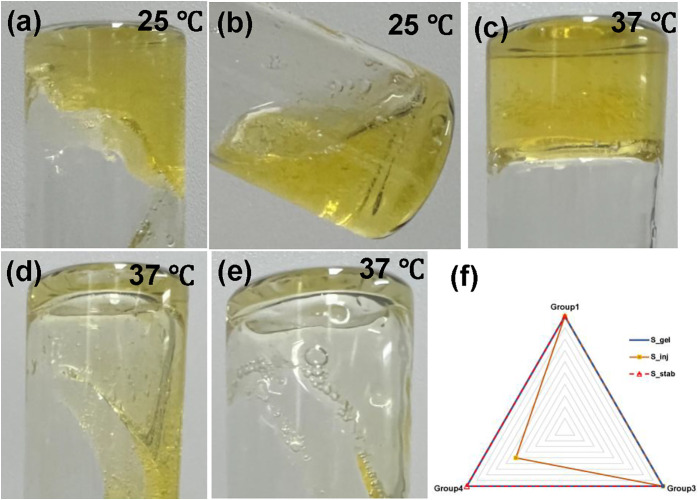
Gelation behavior and stability of CS/β-GP/HEC hydrogels at 25 °C and 37 °C. (a) At 25 °C, G2 exhibited poor fluidity. (b) In contrast, G1 and G3 remained in a liquid state with good fluidity at the same temperature. (c) Upon heating to 37 °C, G1 and G3 formed stable gels that did not flow upon inversion and showed negligible adherence to the vial wall. (d) G4–G6 displayed varying degrees of wall adherence at 37 °C. (e) G7 and G8 presented a semi-gelled state with visible flow upon inversion at 37 °C. (f) Radar chart summarizing the relative scoring profiles of candidate formulations (G1, G3, and G4).

The thermogelation behavior and gelation time of the CS/β-GP/HEC hydrogels were systematically evaluated (Table 3, Fig 1), with successful gelation defined as the absence of flow within 30 s upon vial inversion at 37 °C. At 25 °C, most formulations exhibited acceptable pre-injection flowability (Fig 1b), except for G2, which showed partial pre-gelation and poor fluidity (Fig 1a). Upon heating to 37 °C, gelation times varied markedly (Table 3). G1 and G3 rapidly formed homogeneous, stable gels (10 and 17 min, respectively) with negligible adherence to the vial wall (Fig 1c). Conversely, G2 gelled exceptionally fast (5 min), G4–G6 exhibited varying degrees of vial-wall adherence with gelation times ranging from 13 to >30 min (Fig 1d), and G7–G8 remained semi-gelled with visible flow even after 30 min (Fig 1e).

**Table 3:** Physical states and injectability of CS/β-GP/HEC hydrogels.

Group	State at 25 °C	State at 37 °C	Gelation time (min)	Injectability
1	Liquid	Gel (non-flowing)	10	Injectable
2	Liquid	Gel (non-flowing)	5	Clogged
3	Liquid	Gel (non-flowing)	17	Injectable
4	Liquid	Gel (non-flowing)	13	Partially clogged
5	Liquid	Partially gelled (flowing)	＞30	Injectable
6	Liquid	Partially gelled (flowing)	＞30	Injectable
7	Liquid	Partially gelled (flowing)	＞30	Injectable
8	Liquid	Partially gelled (flowing)	＞30	Injectable

All formulations appeared as pale yellow liquids at 25 °C. Gelation was defined as the absence of flow within 30 s upon vial inversion at 37 °C. Injectability was evaluated using a 1 mL syringe; formulations were considered injectable if continuous extrusion was achieved without clogging.

Injectability was assessed via continuous extrusion through a 26G needle (0.45 mm inner diameter) at 0.1–0.5 mL/min [16,20]. G1 and G3 demonstrated excellent injectability without clogging. From a compositional perspective, higher β-GP concentrations primarily accelerated gelation kinetics at 37 °C (*S*_*gel*_), which inherently caused the complete needle clogging observed in G2. Conversely, higher HEC concentrations significantly increased the pre-gel viscosity, thereby dictating injectability (*S*_*inj*_) and leading to the partial needle blockage seen in G4. The overall structural stability (*S*_*stab*_), further corroborated by mass loss data (Section 3.3), strongly depended on the synergistic ratio between CS and β-GP. Evaluating the dual requirements of stable gelation at 37 °C and favorable injectability at room temperature, a weighted scoring model (Fig 1f) confirmed that G1 and G3 optimally satisfied the selection criteria for injectable thermosensitive hydrogels, designating them as the primary candidates. For narrative clarity, their advanced rheological and in vitro biological performances are highlighted in the subsequent sections, while the comparative mass loss and baseline microstructural data of all eight initial formulations are detailed in Sections 3.3 and 3.4 to strictly validate this screening rationale [21].

### Dynamic rheological properties of the hydrogels

**Fig 2:**
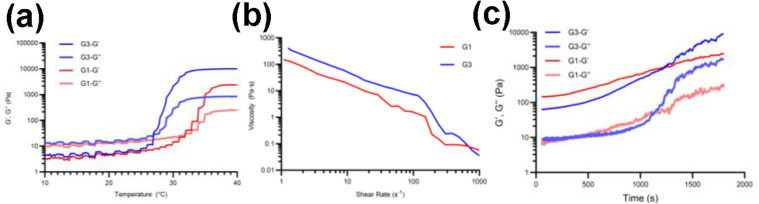
Dynamic rheological properties of the G1 and G3 hydrogel formulations. (a) Temperature sweep profiles showing the evolution of storage (G′) and loss (G″) moduli as a function of temperature. (b) Flow curves of viscosity versus shear rate at 25 °C, demonstrating the shear-thinning behavior of the ungelled formulations. © Isothermal time sweep curves of G′ and G″ at 37 °C, illustrating the gelation kinetics of the hydrogels over time.

The viscoelastic properties and gelation kinetics of the G1 and G3 formulations were characterized using dynamic rheology (Fig 2). Temperature sweep tests revealed that the G3 formulation exhibited a sharp increase in the storage modulus (G′) with a crossover point (Tgel) at approximately 29.5 °C, whereas the G1 formulation displayed a Tgel of approximately 34 °C (Fig 2a). The final plateau G′ of G3 reached nearly 10^4^ Pa, surpassing that of G1 (~2 × 10^3^ Pa).

Isothermal time sweep tests at 37 °C (Fig 2c) demonstrated a rapid increase in modulus for both formulations, with G3 exhibiting faster gelation kinetics and reaching a stable plateau within 1800 s. Additionally, both G1 and G3 showed shear-thinning behavior at 25 °C, characterized by a continuous decrease in viscosity as the shear rate increased from 1 to 1000 s⁻^1^ (Fig 2b). This rheological profile confirms that the formulations possess both thermo-responsiveness and favorable injectability.

### In vitro mass loss in L-DMEM

**Fig 3:**
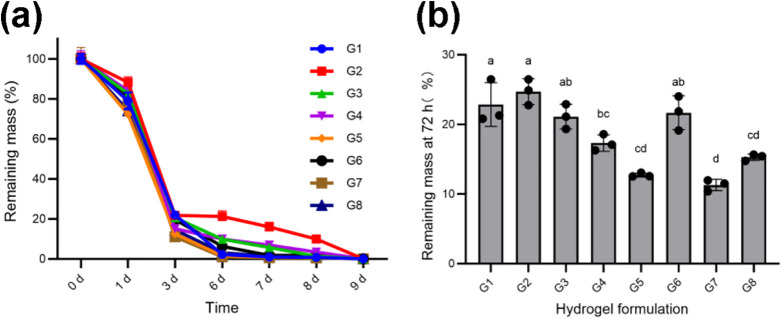
In vitro mass-loss behavior of CS/β-GP/HEC hydrogels in L-DMEM. (a) Time-dependent mass loss of eight hydrogel formulations (G1–G8) over a 9-day incubation period. All groups exhibited a biphasic degradation profile, characterized by rapid mass loss within the first 72 h followed by a slower degradation phase thereafter. (b) Remaining mass (%) at 72 h. Data are presented as mean ± SD (n = 3). Different letters indicate statistically significant differences among groups (one-way ANOVA followed by Tukey’s post hoc test, p < 0.05).

All groups exhibited a time-dependent reduction in mass when incubated in L-DMEM, following a biphasic pattern: rapid mass loss during the first 0–72 h, followed by a slower decrease after 72 h [22,23]. As shown in Fig 3a, the rapid initial phase resulted in substantial mass reduction. Based on the remaining mass at 72 h, the calculated mass loss for G1 was approximately 77.0% (leaving ~23.0% remaining mass). G3 exhibited a mass loss of approximately 79.0% (~21.0% remaining mass), and G8 showed a mass loss of approximately 85.0% (~15.0% remaining mass). By day 9, all groups approached near-complete mass loss under the present incubation conditions. As shown in Fig 3b, significant differences in remaining mass at 72 h were observed among groups (p < 0.05), with G1, G2, G3, and G6 exhibiting significantly higher remaining mass compared to G5 and G7. These results indicate that while the formulations modulated the early mass-loss rate, near-complete mass loss occurred for all groups under the present incubation conditions.

### Microstructural differences (light microscopy/H&E/SEM)

**Fig 4:**
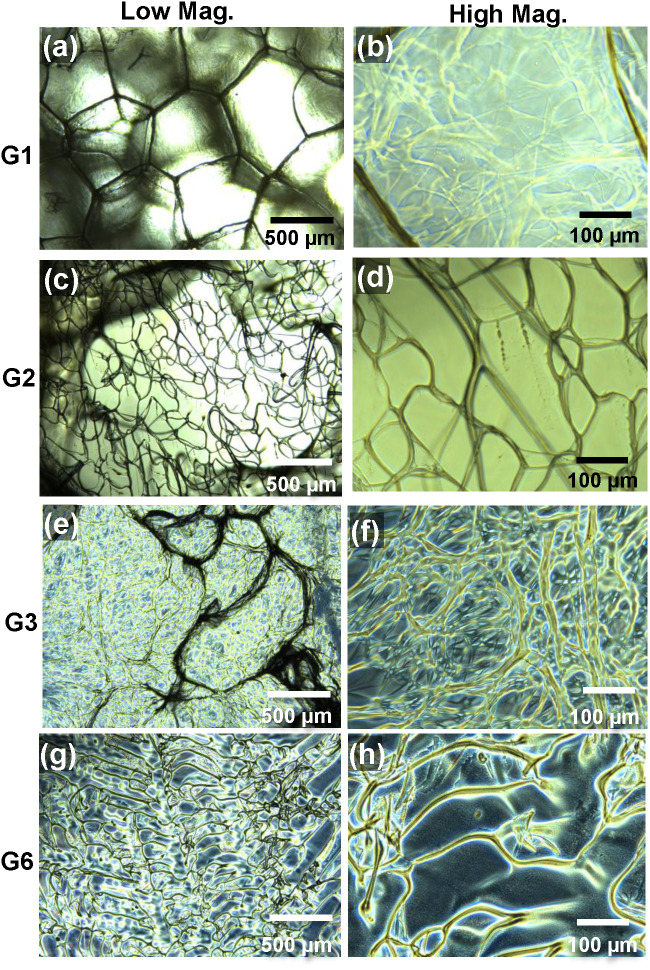
Optical microscopy observation of the microstructures of representative CS/β-GP/HEC hydrogels. (a, b) G1; (c, d) G2; (e, f) G3; and (g, h) G6. For each group, the left column presents low-magnification images showing the overall macroscopic pore architecture, while the right column presents high-magnification images detailing the local polymer network skeletons. Scale bars: 500 μm for the low-magnification images (left column) and 100 μm for the high-magnification images (right column).

Phase-contrast microscopy revealed pronounced differences in network skeleton and pore architecture among the formulations. G1 exhibited honeycomb-like large pores formed by primary branches, with fibrous secondary branches interwoven within each large pore to create numerous small pores (Fig 4a, b). G2 displayed irregular large pores rather than clear honeycomb structures (Fig 4c, d). G3 showed denser secondary and tertiary branching and an increased abundance of small pores, built upon irregular large pores (Fig 4e, f). Other groups exhibited elongated pores, mesh-like structures, or spiny branching patterns (e.g., G6; Fig 4g, h), indicating that the three compositional factors synergistically influenced network formation.

**Fig 5:**
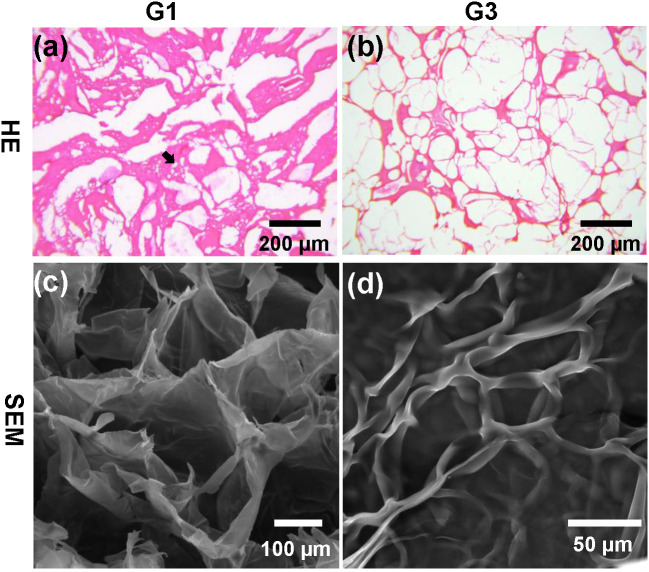
Microstructural characterization of the selected candidate formulations. (a, b) Hematoxylin and eosin (H&E) staining revealing the cross-sectional pore architecture and polymer skeletons of G1 and G3, respectively. (c, d) Scanning electron microscopy (SEM) structural morphology detailing the highly interconnected pore walls of G1 and G3, respectively. Note: Different magnifications were utilized in SEM imaging to optimally capture the distinct characteristic pore sizes and intrinsic structural specificities of each formulation.

Consistent with the aforementioned comprehensive screening, H&E staining of the optimized candidates revealed that G3 possessed a supporting framework of thicker primary skeletons with fine secondary branches forming an open porous network (Fig 5b). In contrast, G1 mainly exhibited lamellar skeletons with heterogeneous pore sizes and a relatively denser structure (Fig 5a). SEM analysis further confirmed that both candidates formed three-dimensional porous networks but differed in pore-wall morphology and pore regularity (Fig 5c, d).

### Cytocompatibility and cellular morphology

**Fig 6:**
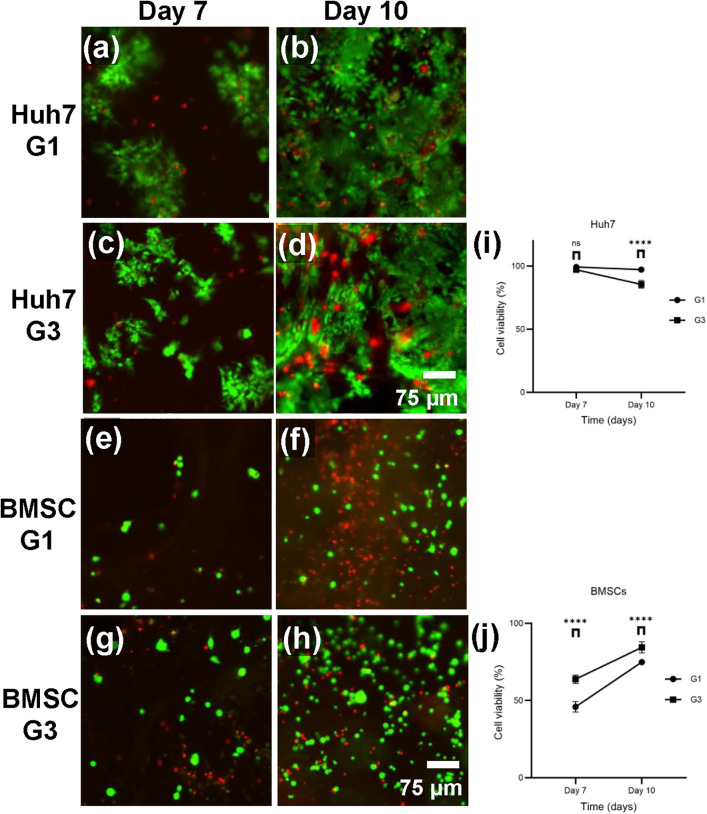
Cell viability of encapsulated Huh7 cells and BMSCs in candidate hydrogels evaluated via Live/Dead staining. The hydrogels include G1 and G3. (a–d) Representative high-magnification images of local regions showing morphology of Live/Dead-stained Huh7 cells in G1 hydrogels at Days 7 and 10 (a, b), and in G3 hydrogels at Days 7 and 10 (c, d). (e–h) Representative local views of Live/Dead-stained BMSCs in G1 hydrogels at Days 7 and 10 (e, f), and in G3 hydrogels at Days 7 and 10 (g, h). Live cells are indicated by green fluorescence (Calcein-AM) and dead cells by red fluorescence (PI). Scale bar: 75 μm. (i, j) Quantitative analysis of cell viability over a 10-day period for Huh7 cells and BMSCs, respectively, calculated from multiple macroscopic randomized fields. Data are expressed as mean ± SD (n = 3). Statistical significance between G1 and G3 candidate formulations at corresponding time points is denoted as **** *p* < 0.0001, and ‘ns’ indicates no significant difference.

Following the selection of optimal formulations, the G1 and G3 hydrogel candidates were evaluated for cytocompatibility using Huh7 cells and BMSCs over a 10-day period. The Live/Dead assay revealed robust cell survival in both groups, characterized by predominant Calcein-AM green fluorescence and minimal PI-positive red signals (Fig 6). For Huh7 cells, the green fluorescence density increased notably from Day 7 to Day 10, particularly in the G3 group (Fig 6a–d, i). Similarly, BMSCs exhibited progressive proliferation, with the G3 formulation supporting a visibly higher cell density by Day 10 (Fig 6e–h, j). The scarcity of red fluorescence confirmed the low cytotoxicity of these hydrogel scaffolds.

**Fig 7:**
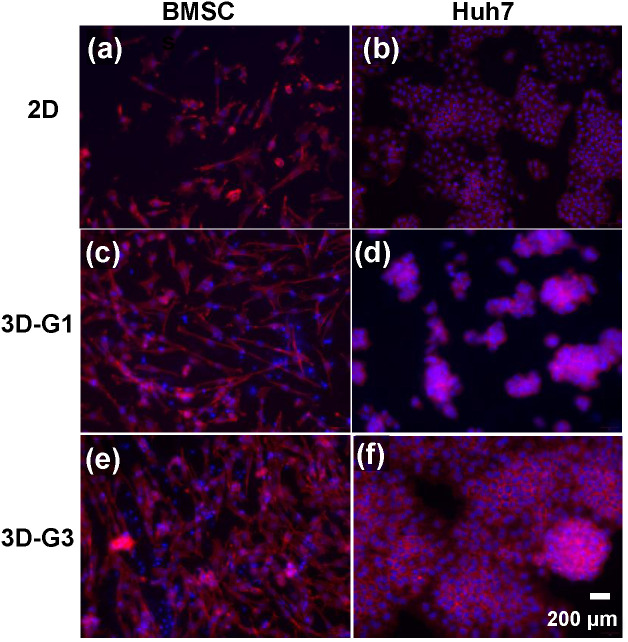
Cellular morphology and spatial organization of BMSCs and Huh7 cells in 2D and 3D culture environments after 7 days of culture. Cytoskeletal F-actin was stained with Phalloidin (red), and nuclei were stained with DAPI (blue). (a, b) Traditional 2D culture control showing flattened BMSCs and monolayer clusters of Huh7 cells. (c, d) Cells encapsulated in the 3D-G1 hydrogel, exhibiting initial spatial spreading and aggregation. (e, f) Cells encapsulated in the 3D-G3 hydrogel, where BMSCs formed a highly interconnected 3D cellular network (e), and Huh7 cells self-assembled into dense, compact multicellular spheroids (f). Scale bar = 200 μm (applies to all images).

Phalloidin/DAPI staining at Day 7 further detailed the morphological differences between standard 2D and 3D culture environments (Fig 7). In the 2D control, BMSCs displayed a flattened, spindle-shaped morphology with prominent actin stress fibers, while Huh7 cells grew in typical monolayer clusters (Fig 7a, b). Within the 3D hydrogels, the encapsulated cells exhibited architectures more representative of physiological conditions. BMSCs in the G3 group (Fig 7e) developed a dense, interconnected cellular network with multi-directional spreading, demonstrating more extensive 3D spatial organization compared to the G1 group (Fig 7c). Meanwhile, Huh7 cells in the G3 group (Fig 7f) self-assembled into dense multicellular spheroids with high cell-cell contact, characteristic of typical 3D tumor models, compared to the looser aggregates observed in G1 (Fig 7d). These findings indicate that the optimized G3 hydrogel provides an effective structural environment for supporting cell-specific spatial organization and 3D growth.

### Comparison of cell proliferation in 3D hydrogels and 2D culture

**Fig 8:**
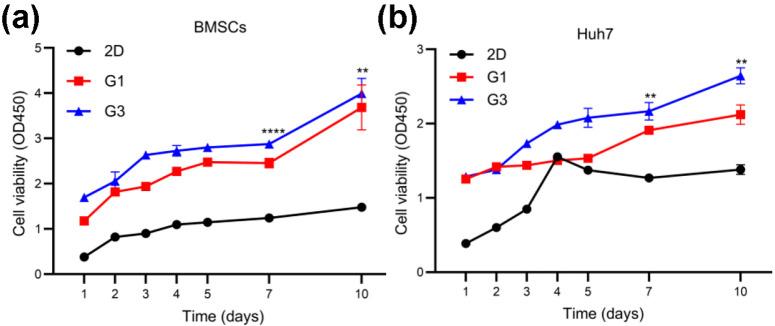
Quantitative evaluation of cell proliferation in 2D monolayer cultures and 3D hydrogels (G1 and G3) over a 10-day period using the CCK-8 assay. (a) Metabolic activity profiles of BMSCs. (b) Metabolic activity profiles of Huh7 cells. Data are presented as mean ± SD (n = 3). Statistical significance between specific groups is denoted as **p < 0.01 and ****p < 0.0001.

Cell proliferation within the 3D hydrogels (G1 and G3) and conventional 2D monolayer cultures was quantified using the CCK-8 assay over a 10-day period (Fig 8). For BMSCs (Fig 8a), cell metabolic activity in both 3D groups increased progressively from Day 1 to Day 10. Notably, the metabolic activity in the G3 group was significantly higher than that in the G1 group at Day 7 (p < 0.0001) and Day 10 (p < 0.01). In contrast, the 2D control group exhibited significantly lower proliferation rates compared to the 3D groups throughout the study. For Huh7 cells (Fig 8b), the metabolic activity in the 2D control group reached a plateau by Day 4 and remained stable or slightly declined thereafter. However, Huh7 cells encapsulated in both G1 and G3 hydrogels demonstrated continuous growth up to Day 10. At the final time point (Day 10), the metabolic activity of Huh7 cells in the G3 group was significantly higher than that in both the G1 group (p < 0.01) and the 2D control group.

## Discussion

### Synergistic effects of components on macroscopic gelation and rheological properties

An injectable thermosensitive hydrogel must remain in a liquid, operable state at room temperature and rapidly form a shape-stable gel at body temperature [21]. In this study, our rheological characterization confirmed that both G1 and G3 formulations exhibited distinct thermogelation profiles suitable for clinical handling. Notably, the G3 formulation achieved a storage modulus (*G*′) of nearly 10⁴ Pa, significantly higher than that of G1 (∼2 × 10^3^ Pa). This enhanced stiffness, coupled with a lower *T*_*gel*_ (29.5 °C), suggests that G3 provides more immediate and robust structural support upon injection. As suggested in previous studies, increased chain entanglement and the presence of HEC promote the formation of a more continuous network, thereby improving shape stability [21,24].

The selection of these formulations represents a balance between gelation kinetics and injectability. Increasing the β-GP concentration typically accelerates gelation but may increase the risk of needle clogging [24,25]. Our findings imply that HEC viscosity synergizes with β-GP to modulate the formulation’s resistance to flow and its initial structural integrity [26,27]. Consequently, the trade-off between “fast gelation” and “injectability” must be balanced according to the specific application [28]. The selection of medium-viscosity CS (200–400 mPa·s) and HEC was intended to mimic the soft mechanical microenvironment of visceral tissues, where appropriate stiffness provides critical physical cues for cells.

### Interpretation and methodological boundaries of mass loss

In L-DMEM without lysozyme, the measured mass change is characterized as *in vitro* mass loss rather than enzymatic degradation of the polymer backbone [29]. Initial changes likely involve swelling, dissolution of soluble components, and progressive loosening of the polymeric network [30,31]. Although the acellular mass loss data suggest a structural lifespan of approximately 9 days, it is important to distinguish this from the dynamic environment of actual cell culture. In the presence of cells, the synthetic CS/β-GP/HEC framework does not simply disappear; rather, it undergoes cell-mediated remodeling and bio-integration.

Encapsulated cells, particularly BMSCs, actively interact with the scaffold and secrete their own extracellular matrix (ECM) as the synthetic framework gradually loosens. This creates a “living” hybrid microenvironment in which the newly synthesized biological matrix compensates for the loss of the synthetic polymer, thereby providing sustained 3D support beyond the initial acellular lifespan. Our observation that BMSC viability and density in the G3 group at Day 10 were significantly higher than those in the 2D control (as shown in the proliferation data) provides strong evidence for this sustained 3D environment. If the cells had settled into a 2D monolayer by Day 10 due to scaffold failure, their proliferation would have been restricted by contact inhibition, leading to a growth plateau similar to that of the 2D control. Instead, the continued robust growth in G3 confirms that the cells remain in a supportive, multi-dimensional niche. Although the specific morphological transition during this bio-remodeling process warrants further investigation through long-term ECM staining, our current findings validate the functional stability of this system for 3D culture applications.

### Systematic scoring, main effects, and candidate selection

Although a formal statistical regression model was not the primary focus of this study, our experimental matrix systematically evaluated the main effects and synergistic interactions of CS, β-GP, and HEC to balance gelation kinetics (*S*_*gel*_), injectability (*S*_*inj*_), and structural stability (*S*_*stab*_). Specifically, β-GP concentration was identified as the primary factor driving gelation behavior (*S*_*gel*_), and our rheological data confirmed that an optimal interaction with CS is essential to trigger the sol-gel transition crossover at 37 °C. Meanwhile, HEC concentration predominantly affected injectability (*S*_*inj*_) by modulating initial viscosity and shear-thinning properties. Structural stability (*S*_*stab*_) relied heavily on the interaction between CS and β-GP, which determined the density of thermosensitive physical crosslinking. Ultimately, the overall performance (*S*_*total*_) resulted from the synergistic effects of these multifactorial interactions.

The weighted scoring approach was instrumental in applying these factorial interactions to identify optimal formulations from the complex 2 × 2 × 2 factorial design. By establishing hard exclusion criteria (no flow at 37 °C and passability through a needle), we effectively filtered out formulations with poor mechanical stability (G5–G8) or operational failure (G2). Among the remaining candidates, G1 and G3 achieved the highest aggregate scores by striking a precise balance between gelation speed (*S*_*gel*_) and injectability (*S*_*inj*_), unlike G4, which was penalized for partial clogging. This quantitative approach ensures that the selected candidates possess the requisite handling characteristics for practical clinical applications.

These multifactorial interactions ensure that the final formulations provide an ideal microenvironment for cell growth and recruitment, as demonstrated by our newly added rheological and morphological findings. Biological validation confirmed that these optimized 3D scaffolds (G1 and G3) offer significant advantages over conventional 2D culture. The superior cell proliferation and spatial organization observed in these groups demonstrate that the screened physical parameters successfully established a biomimetic architecture that facilitates mass transport and protects cells from mechanical shear, highlighting the high potential of these formulations in 3D cell culture and tissue engineering.

### Microstructural Differences and Potential Origins

Light microscopy, H&E staining, and SEM revealed distinct differences in skeletal morphology and pore architecture between the two hydrogel candidates. G3 exhibited a thicker primary framework with a porous network characterized by fine, interwoven secondary branches. In contrast, G1 displayed predominantly lamellar skeletons with heterogeneous pores and an overall denser structure. **Given that G1 and G3 differ solely in their β-GP formulation (36% vs. 5.6%, respectively)**, these structural differences strongly suggest that β-GP concentration dictates the strength of intermolecular interactions and the degree of network condensation, thereby governing skeleton formation and pore architecture [24]. This study primarily relied on representative images; quantitative image analysis (e.g., pore-size distribution, porosity/area fraction) would enhance reproducibility and enable more direct comparisons.

### Biological performance: Morphological adaptation, rationale, and limitations

To validate the versatility of the hydrogels for different tissue models, Huh7 cells and BMSCs were deliberately selected. Huh7 cells represent an epithelial-derived model that is highly dependent on cell-cell contact, making them ideal for evaluating tumor spheroid formation [32]. Conversely, BMSCs represent a mesenchymal stromal model that is highly sensitive to mechanical cues and critical for tissue regeneration.

Live/Dead staining confirmed that both cell types maintained high viability. Importantly, CCK-8 assays demonstrated that cellular proliferation was robustly sustained in 3D cultures, bypassing the contact inhibition observed in 2D cultures, where growth plateaued by Day 4 (Huh7) or remained significantly lower (BMSCs). While these results highlight the advantages of the 3D hydrogel, it is important to carefully interpret the inherent dimensional differences between these systems. In our experimental design, following standard evaluation protocols for biomaterial-cell composites, cells were seeded at an equivalent initial cell concentration (cells/mL) rather than matching the total surface area. Technically, precisely equalizing the vast internal surface area of a highly porous, swollen 3D hydrogel with a flat 2D substrate remains highly challenging in current *in vitro* setups, as physical parameters such as dynamic surface area, porosity, and structural stiffness are fundamentally coupled in 3D matrices [33–34]. Consequently, the superior cell proliferation and morphological adaptation observed in the 3D scaffolds are likely a synergistic outcome: driven both by the significantly larger available surface area—which delays contact limitation—and by the inherent 3D microenvironment that provides physiological spatial cues and proper mechanotransduction. Notably, although *in vitro* acellular degradation showed rapid mass loss by Day 9, the encapsulated cells did not settle into a 2D monolayer. Instead, they underwent cell-mediated microenvironment remodeling. Beyond simple survival, Phalloidin/DAPI staining revealed that the hydrogel matrix supported cell-specific morphological adaptations. During the 10-day culture, the hydrogel provided initial structural support (e.g., the 104 Pa stiffness in G3) while gradually loosening sufficiently to permit cellular aggregation [35] and potential extracellular matrix (ECM) secretion. The formation of Huh7 spheroids in G3 highlights the hydrogel’s ability to facilitate self-assembly, closely mimicking the *in vivo* 3D tumor microenvironment. Similarly, the ability of BMSCs to extend filopodia and form multi-directional spatial networks in G3 suggests that the porous architecture provides an ideal mechanical niche for sustained 3D support.

Despite these promising results, we acknowledge certain methodological limitations. While Huh7 cells and BMSCs successfully demonstrated the scaffolding capability for epithelial aggregation and mesenchymal spreading, relying on these two specific cell lines limits the immediate generalization to all “diverse tissue models.” The complexity of *in vivo* tissue engineering often requires the presence of multiple supporting cell types. Furthermore, although our data validates short-term 3D morphological adaptation, evaluating the long-term, tissue-specific functional differentiation of encapsulated cells remains a limitation. Additionally, while the sample size for the *in vitro* assays in this study (n = 3) was sufficient to identify statistically significant trends, this relatively small cohort limits absolute statistical power. Future studies should incorporate primary cells, patient-derived organoids, or multicellular co-culture systems, alongside long-term functional assays, quantitative image analysis, advanced mathematical modeling to decouple the effects of surface area from 3D topography, and larger sample sizes, to fully substantiate the broad translational potential of these optimized hydrogel formulations.

## Conclusions

In this study, a systematic factorial design and multi-parameter evaluation were employed to optimize injectable CS-β/HEC thermosensitive hydrogels. By elucidating key multifactorial interactions, two macroporous candidates (G1 and G3) were identified. Rheological characterization confirmed their excellent shear-thinning injectability and robust mechanical strength, with G3 achieving a storage modulus of nearly 10^4^ Pa. Biologically, these scaffolds supported a dynamic microenvironment that accommodated cell-mediated remodeling. This enabled BMSCs to form interconnected 3D spatial networks and Huh7 cells to self-assemble into multicellular spheroids, demonstrating structural support and physiological relevance that were significantly superior to those of conventional 2D cultures.

Although long-term in vivo degradation mechanisms and validation across a broader range of primary cell types require further investigation, rational formulation screening remains critical for tailoring pore architecture and mechanical cues to meet the diverse needs of different tissue models [36–38]. The present work provides structured comparative data and robust biological validation, offering a reliable methodological foundation for advancing CS-based thermosensitive hydrogels in 3D cell culture and tissue engineering.

## Supporting information

S1 File Data. (XLSX)
